# Efficacy of 4.0 mg versus 0.4 mg Folic Acid Supplementation on the Reproductive Outcomes: A Randomized Controlled Trial

**DOI:** 10.3390/nu13124422

**Published:** 2021-12-10

**Authors:** Renata Bortolus, Francesca Filippini, Sonia Cipriani, Daniele Trevisanuto, Francesco Cavallin, Giovanni Zanconato, Edgardo Somigliana, Elena Cesari, Pierpaolo Mastroiacovo, Fabio Parazzini

**Affiliations:** 1 Office for Research Promotion, Verona University Hospital, 37134 Verona, Italy; renata.bortolus@aovr.veneto.it (R.B.); francesca.filippini83@gmail.com (F.F.); 2 Department of Woman, Newborn and Child, Fondazione Istituto di Ricovero e Cura a Carattere Scientifico Ca’ Granda Ospedale Maggiore Policlinico, 20122 Milan, Italy; sonia.cipriani@policlinico.mi.it (S.C.); dadosomigliana@yahoo.it (E.S.); 3 Department of Woman’s and Child’s Health, University of Padua, 35128 Padova, Italy; daniele.trevisanuto@unipd.it; 4 Independent Statistician, 36020 Solagna, Italy; cescocava@libero.it; 5 Department of Surgery, Odontostomatology and Maternal and Child Health, University of Verona, 37134 Verona, Italy; giovanni.zanconato@univr.it; 6 Department of Clinical Sciences and Community Health, University of Milan, 20122 Milan, Italy; 7 Department of Obstetrics and Gynaecology, Sant’Antonio Abate Hospital, 21013 Gallarate, Italy; elenacesari@gmail.com; 8 International Centre on Birth Defects—ICBD, 00195 Rome, Italy; pierpaolo.mastroiacovo@gmail.com

**Keywords:** folic acid, randomized controlled trial, reproductive outcomes, spontaneous abortion, preterm delivery

## Abstract

Folic acid (FA) supplementation prevents neural tube defects (NTDs), but the effects on other reproductive outcomes are unclear. While common recommendation is 0.4 mg/day in addition to regular nutrition, the most appropriate dose of FA is still under debate. We investigated the effects of a higher dose of periconception FA on reducing adverse reproductive outcomes. In this multicenter double-blind randomized controlled trial (RCT), 1060 women (aged 18–44 years and planning a pregnancy) were randomly assigned to receive 4.0 mg or 0.4 mg of FA daily. The primary outcome was the occurrence of congenital malformations (CMs). A composite outcome including one or more adverse pregnancy outcomes was also evaluated. A total of 431 women had a natural conception within 1 year. The primary outcome occurred in 8/227 (3.5%) women receiving 4.0 mg FA and 9/204 (4.4%) women receiving 0.4 mg FA (RR 0.80; 95%CI 0.31 to 2.03). The composite outcome occurred in 43/227 (18.9%) women receiving 4.0 mg FA and 75/204 (36.8%) women receiving 0.4 mg FA (RR 0.51; 95%CI 0.40 to 0.68). FA 4.0 mg supplementation was not associated with different occurrence of CMs, compared to FA 0.4 mg supplementation. However, FA 4.0 mg supplementation was associated with lower occurrence of other adverse pregnancy outcomes.

## 1. Introduction

Folic acid (FA) supplementation before conception and in early pregnancy is recommended to women planning a pregnancy to reduce the risk of neural tube defects (NTDs). This protective effect has led to mandatory flour fortification with FA in several countries (such as United States, Canada, Chile, South Africa, and Australia) and to recommendation of daily FA supplement in women planning a pregnancy (such as United States, Canada, Australia, and Europe, including Italy). In 2015, a Cochrane review [1] confirmed that FA, alone or in combination with vitamins and minerals, prevents the first and second time occurrence of NTDs, but did not find a clear effect on other birth defects (namely cleft palate, cleft lip, congenital cardiovascular defects) as previously suggested [2,3]. Moreover, the available literature offers an uncertain impact of FA supplementation on fertility and prevention of other adverse reproductive outcomes [4,5,6,7,8,9,10,11,12,13,14,15,16]. Observational studies indicated an association between greater level of folate during pregnancy and higher birthweight [6] and fewer cases of small for gestational age (SGA) [7,8] and preterm delivery [9], although such findings were not reported in other investigations [10,11].

To date, the relationship between folate insufficiency and the onset of placenta mediated diseases, such as spontaneous abortion, preterm birth, fetal growth restriction, preeclampsia [13,14], abruptio placentae [15] and stillbirth [2,16] is not fully understood, and the issue of the most suitable dose of FA is still open [17,18].

Currently, the most common public health recommendation in Europe, including Italy, to prevent the occurrence of NTDs, in healthy women of childbearing age planning a pregnancy, or if pregnancy is a possibility, is to take a FA supplement containing 0.4 mg/day, at least one month before conception until 8–12 weeks of pregnancy [19]. The standard recommendation for recurrence prevention of NTDs, and for occurrence prevention in epileptic and diabetic mothers, is to take a higher dose of FA, 4–5 mg/day. Compulsory food fortification with FA has not yet been established in the European countries, including Italy, and enhance sources of folate are obtainable only from FA supplements, voluntary fortification, and consumption of foods rich in folates such as vegetables and fruits.

A review of efficacious doses [20] suggested a dose-dependent effect but further investigation failed to find an optimal folate status in the European population [21]. Furthermore, real-life data suggest that many pregnant women use high doses of FA, up to 5 mg/day and even more, in several European countries [22,23].

Overall, population concerns about FA were mainly associated with the effect of masking pernicious anemia due to B12 deficiency, the possible effects on cancer and potential changes in offspring neurodevelopment and epigenetic modifications [17,18,24,25].

In this randomized controlled trial (RCT), we evaluated the effect of periconception FA supplementation of 4.0 mg/day compared to 0.4 mg/day standard dose on the occurrence of congenital malformations (CMs). A composite outcome including one or more adverse pregnancy outcomes was also evaluated. We hypothesized that higher intake of FA may be associated with the reduction of adverse pregnancy outcomes. The doses of FA were based on evidence of efficacy, recommendations regarding occurrence and recurrence of NTDs [26], and studies on other adverse pregnancy outcomes [27,28].

## 2. Materials and Methods

### 2.1. Trial Design and Participants

In this multicenter, 2-arm, double-blind RCT, we compared FA supplementation using 4.0 mg/day vs. 0.4 mg/day, from randomization until the 12th gestational week, in women of childbearing age (18–44 years old) who planned a pregnancy within 12 months (ClinicalTrials.gov: NCT01244347; EudraCT: 2008-004334-25). In the absence of pregnancy after 12 months, women discontinued FA supplementation and ended the participation in the study.

The trial started in Italy in July 2009 and was stopped in February 2014 due to difficulties in subject recruitment. It was conducted at 33 randomization centers, including 13 maternity hospitals, 11 family planning services, 3 assisted reproduction centers, and 6 general practitioners.

Women were not eligible if they: were pregnant; did not plan a pregnancy; were younger than 18 or older than 44 years; were planning to move where the study was not implemented; did not understand Italian; did not own a telephone; had a diagnosis of epilepsy, diabetes, obesity (Body Mass Index (BMI) ≥ 30 km/m^2^) or megaloblastic anemia; had suffered from cancer or a serious disease (e.g., Crohn disease, rheumatoid arthritis, ulcerative colitis); were being treated with antifolates (such as methotrexate) or had recently been treated with it; abused (4–6 drinks/day) or had abused (4–6 drinks/day in the previous 12 months) alcohol; were vegetarian; had a previous pregnancy with NTD or any other congenital structural birth defects; were affected or had a partner affected by NTD; had a relative affected by NTD; had a family medical history of breast cancer or colorectal cancer; had a personal or family history of a hereditary syndrome such as familiar adenomatous polyposis or hereditary nonpolyposis colorectal cancer; reported allergy to FA or presented contraindications to FA use; took defined dosages of FA. Most of the exclusion criteria referred to conditions for which FA supplementation at doses of 4–5 mg/day is recommended, so it would have been unethical to randomize these patients to 0.4 vs. 4 mg/day. Some exclusion criteria related to the feasibility of follow-up.

At the enrolment visit, preconception counselling was offered to women according to standard guidelines [29], to identify and decrease the impact of biomedical, behavioral and social risk factors (such as previous adverse reproductive outcomes, lifestyle, maternal conditions or chronic diseases) that might affect health and pregnancy outcomes. Preconception counselling included a visit with the local investigator in which many aspects of pregnancy were discussed. The purpose was to identify any risk factors (such as medical conditions, obesity, use of medications, tobacco, alcohol or drug exposure, family and reproductive history) and to promote protective interventions for a healthy pregnancy (such as recommended immunizations and laboratory tests). Maternal characteristics and medical and obstetric information were recorded. Dietary folate intakes were not assessed.

Eligible women who consented to participate in the trial were randomized. In case of pregnancy, women were interviewed by phone at 16 weeks of gestation, 24 weeks of gestation, and after delivery. The interview was performed by a trained Health Care Provider (HCP) using a structured form and aimed to assess the pregnancy outcome. The health status of livebirths was evaluated by a trained HCP through a phone interview with the pediatrician (at 1, 3 and 12 months of age) and an interview with the parents (at 12 months of age) using a predefined form.

Moreover, clinical research staff obtained data on study outcomes with the collaboration of the investigators and clinical centers. Quality control of enrolment and verification of protocol adherence were performed by the General Coordination Center at the Verona University Hospital.

An independent Data Safety Monitoring Board monitored the trial. The authors guarantee the accuracy and completeness of the data and analyses.

### 2.2. Randomization and Trial-Group Assignment

Eligible women who consecutively referred to the participating centers during the study period were invited to participate. After providing informed consent, eligible women were randomly assigned (in a 1:1 ratio) to receive 4.0 mg (treatment under investigation) or 0.4 mg (reference treatment) of FA daily. The study was double-blinded, as neither the participants nor the investigators and research staff were aware of the treatment assignments. The randomization was stratified according to participating center and maternal age group (<30 years, 30–34 years, >34 years), and the randomization code was generated by a web-based randomization system (IPT Srl, Rome, Italy). The FA tablets, containing vitamin B9 (pteroylglutamic acid), were manufactured by Pierrel Research IMP Srl and were packed, labelled, stored and distributed by the Pharmaceutical Service of Verona University Hospital. The tablets of 4.0 and 0.4 mg of FA were identical with respect to size, thickness and appearance.

Participants were instructed to take only the supplement given by the trial, one tablet of FA every day, and to interrupt at 12 weeks of gestation if conception occurred, providing feedback to the clinical center. Every 4 months, participants referred to the randomization center for a check on pregnancy status and to return any unused tablet, to receive a new supply and to report any side effects. Compliance with FA supplementation was evaluated at the randomization center by counting the tablets in the participant’s box.

### 2.3. Outcome Measures

The original protocol included the occurrence of CMs as primary outcome measure. Due to difficulties in recruitment (see below), a composite outcome (including one or more of these adverse pregnancy outcomes: spontaneous abortion, intrauterine fetal death, preeclampsia, abruptio placentae, SGA, preterm delivery, major CMs) was also considered as an outcome measure of the trial [30].

Major CMs were defined as structural changes that could have significant medical, social or cosmetic consequences for the affected individual, and typically require medical intervention. Minor CMs were structural changes that posed no significant health problem in the neonatal period, with limited social or cosmetic consequences for the affected individual according to EUROCAT [31].

Spontaneous abortion was defined as the loss of clinically recognized pregnancy less than 22 weeks gestation; fetal death was defined as intrauterine death from 22 weeks gestation according to the World Health Organization.

Preeclampsia was defined as systolic blood pressure ≥ 140 mmHg or diastolic blood pressure ≥ 90 mmHg presenting after 20 weeks gestation in a previously normotensive woman combined with > 300 mg protein per 24-h urine collection or protein:creatinine ratio >30 mg/mmol according to NICE [32].

Abruptio placentae was defined as premature separation of a normally located placenta from the uterine wall that occurred before the delivery of the fetus.

SGA was defined as an infant with a birthweight under the 10th percentile for gestational age [33,34]. Preterm delivery was defined as gestational age < 37 weeks.

### 2.4. Adverse Events and Adherence

Any untoward medical occurrence—in a subject receiving a medicinal product which did not necessarily have a causal relationship with this treatment—were considered as adverse events. Adverse events were assessed and recorded during each visit at the randomization center up to 12 weeks of gestation if participants conceived, or to 12 months in the absence of pregnancy.

Adherence was assessed by interviewing the participants and by counting the tablets that were returned by participants at each visit. Adherence was deemed as good if the reported intake of tablets in fully treated women was 70% or more of the total number supposed to be taken between randomization date and 12th week of gestation.

### 2.5. Statistical Analysis

The study started as a combined effort by research groups but encountered difficulties in subject recruitment and was unable to achieve the estimated enrolment of 5000 subjects (EudraCT number 2008-004334-25, ClinicalTrials.gov number, NCT01244347) and 1000 pregnancies [30]. In the original protocol, sample size was calculated using CMs as primary outcome. However, given the growing interest in the effect of FA supplementation on other pregnancy outcomes and the difficulties in recruitment, we added a composite outcome including one or more adverse pregnancy outcomes (list in Section 2.3) [30]. The expected sample size of 1000 pregnancies [30] could not be achieved due to the low rate in patient recruitment, hence enrolment was stopped when 505 pregnancies were diagnosed. This sample size allowed identification an absolute reduction in the composite outcome of about 11% or more, with a power of at least 80% and a type I error of 5%, when assuming a frequency of the composite outcome of 30% or less in the 0.4 mg FA group, considering an attrition rate of 10%. Statistical analysis adopted an intention-to-treat basis and did not include any interim analyses.

All researchers involved in the analyses were masked to the allocation group.

Categorical variables were compared using Chi-square test or Fisher’s exact test, while continuous variables using Student *t*-test. The adjusted effects of the treatment on the outcome measures were estimated using linear models and generalized linear models, adjusting for clinically relevant confounders. Effect sizes were reported as risk ratio (RR) or mean difference (MD), with 95% confidence interval (CI). All tests were 2-sided and a *p*-value less than 0.05 was considered statistically significant. Statistical analysis was performed using SAS 9.4 (SAS Institute, Inc, Cary, NC, USA).

## 3. Results

### 3.1. Trial Findings

#### 3.1.1. Trial Participants

**Figure 1:**
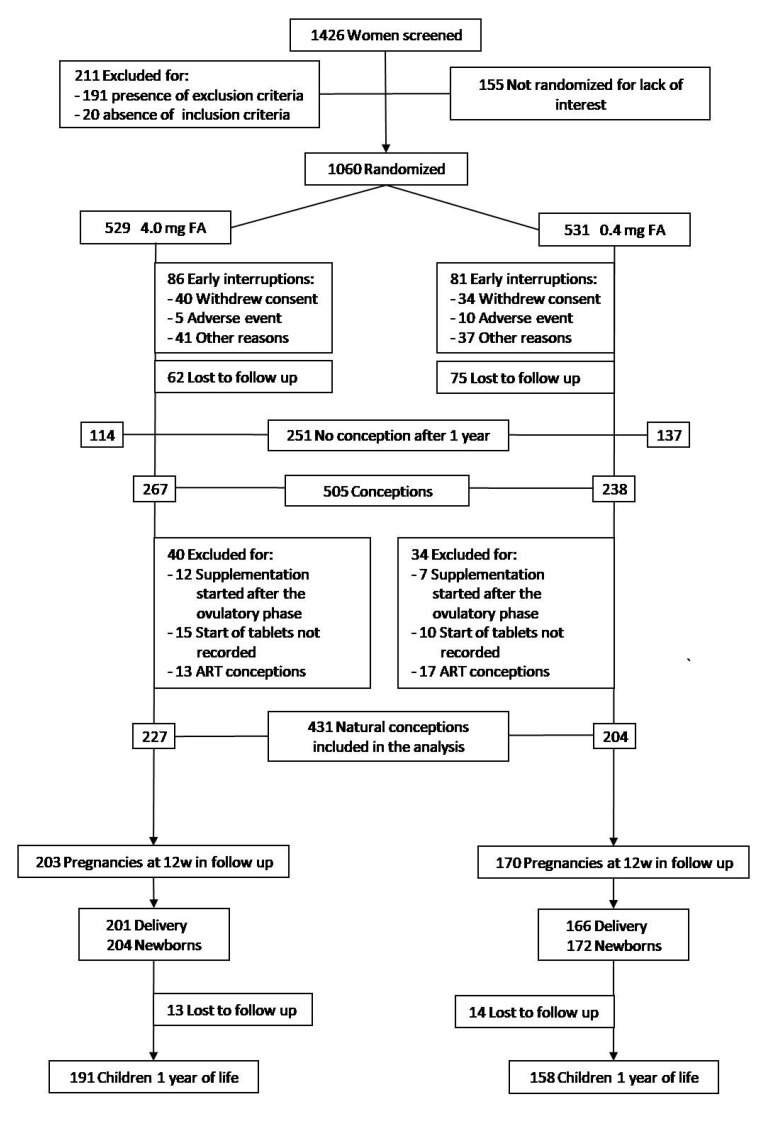
Enrolment, Randomization, and Follow-up.

Figure 1 shows the enrolment, randomization and follow-up of the participants.

Of 1426 women who underwent the enrolment visit, 211 (14.8%) were excluded because they did not meet the eligibility criteria. Of 1215 eligible women, 1060 (87.2%) agreed to participate in the trial and were randomized to receive 4.0 mg (529 women) or 0.4 mg/day (531 women) of FA. The two arms were balanced and there were no significant differences with respect to baseline characteristics (Table S1).

After randomization, 74 women (7.0%) withdrew consent and 93 women (8.8%) were removed from the trial due to onset of exclusion criteria, moving house or adverse events; in addition, 137 women (12.9%) were lost to follow-up. Finally, 251 women (23.7%) did not conceive within one year of FA supplementation.

A total of 505 participants conceived. Of these, 44 (4.2%) were excluded because the FA supplementation started after the ovulatory phase or because the start date of tablet intake was not recorded; furthermore, 30 assisted reproductive technology (ART) conceptions (2.8%) were excluded (Figure 1).

The baseline characteristics of 431 women included in the analysis were balanced between groups (Table 1). There were 227 natural conceptions in the 4.0 mg and 204 in the 0.4 mg FA groups (*p* = 0.07).

#### 3.1.2. Study Outcomes

Among 1060 randomized women, there were 431 natural conceptions which were included in the analysis of the outcomes (Table 2).

The primary outcome occurred in 8/227 (3.5%) women receiving 4.0 mg FA and 9/204 (4.4%) women receiving 0.4 mg FA (RR 0.80; 95% CI 0.31 to 2.03). The composite outcome occurred in 43/227 (18.9%) women receiving 4.0 mg FA and 75/204 (36.8%) women receiving 0.4 mg FA (RR 0.51; 95% CI 0.40 to 0.68). Of note, FA 4.0 mg/day supplementation was associated with lower spontaneous abortions (RR 0.56; 95% CI 0.33 to 0.92), SGA (RR 0.42; 95% CI 0.19 to 0.96) and preterm deliveries (RR 0.45; 95% CI 0.21 to 0.98). These findings were confirmed when adjusting for clinically relevant confounders (Table 2).

Other outcome measures are reported in Table 3.

The 4.0 mg FA group showed higher birthweight (mean 3357 g vs. 3213 g) and lower proportion of low birthweight infants (RR 0.39; 95% CI 0.18 to 0.82) compared to 0.4 mg FA group.

After 12th week of gestation, 31 women in the 4.0 mg FA group and 19 in the 0.4 mg FA group continued to use FA. Among 31 women there were one SGA and three preterm deliveries, while among 19 women there were one SGA and two preterm deliveries, without significant differences between the groups. 

Major CMs included anencephaly, atrial septal defect, two cases of cleft lip and palate, esophageal atresia, post-assial polydactyly left foot, tricuspid valve insufficiency and persistent foramen ovale, webbed penis in the 4.0 mg FA group; anencephaly, atrial septal defect, inguinal hernia, persistent foramen ovale, three cases of trisomy 21 and two of undescended testis in the 0.4 mg FA group. Minor CMs occurred in 16/227 (7.0%) women receiving 4.0 mg FA and 17/204 (8.3%) women receiving 0.4 mg FA (RR 0.85; 95% CI 0.44 to 1.63; *p* = 0.62).

Characteristics of delivery and neonatal variables did not significantly differ between the two groups (Table 4).

There was no significant difference in the rates of male and Apgar 5′ > 7 between groups (*p* = 0.11 and *p* = 0.29 respectively).

#### 3.1.3. Adverse Events and Adherence

One serious unrelated or unlikely related adverse event occurred in three participants (0.6%) in the 4.0 mg FA group and in five participants (0.9%) in the 0.4 mg FA group (*p* = 0.49). At least one adverse event occurred in 97 participants (18.3%) in the 4.0 mg FA group and 114 participants (21.5%) in the 0.4 mg FA group (*p* = 0.20). Respiratory infections, urinary tract infections, thyroid diseases and skin diseases were the most common.

Adherence in pregnant women was good in 327/444 (73.6%) women at the 4th month visit, in 237/340 (69.7%) at the 8th month visit and in 223/251 (88.8%) at the 12th month visit, without any differences between the two groups.

## 4. Discussion

This multicenter, double-blind, RCT involving women of childbearing age who planned a pregnancy within 12 months failed to show any advantage of FA 4.0 mg versus 0.4 mg daily supplementation on the occurrence of CMs. However, FA supplementation at a dose of 4.0 mg daily (started before pregnancy to 12th gestational week) was associated with lower occurrence of spontaneous abortion, SGA, preterm delivery, and a composite outcome including adverse pregnancy outcomes.

The debate on the most appropriate dose and timing of FA to reduce adverse pregnancy outcomes appears relevant, as European studies have failed to identify an optimal level of serum folate [21] and highlighted that pregnant women used high doses of FA supplementation in early- to mid-pregnancy [22,23]. Furthermore, a review of efficacious doses [20] suggested a dose-dependent effect on prevention of birth defects. The authors addressed the issue of the efficacy of high FA dosage more comprehensively, evaluating the effect of increasing FA intake on serum folate levels and on the relationship between such levels and the risk of NTDs during pregnancy. They noticed that, from a typical western background serum folate level of 5 ng/mL, an increase of 0.4 mg/day would lower the risk of NTDs by around 36%, while taking 5 mg/day would lower the risk by around 85%.

The two RCTs clearly displaying a decreased risk of NTDs were conducted with 4.0 mg/day [26] and 0.8 mg/day [35] of FA. Furthermore, a nonrandomized study on the recurrence risk of oral clefts reported a risk reduction of 66% using a periconception FA supplementation of 10 mg/day [36]. Another study indicated that only a high dose (6 mg/day) of FA reduced the risk of oral clefts by 25% but not a smaller dose (<1 mg/day) [37], and a case-control study on Down syndrome reported a reduced risk of 60% after the preconception use of a high dose of FA (6–9 mg/day) [38].

Moreover, several studies provided inconclusive findings about the effect of dose and timing on adverse pregnancy outcomes [4,5,6,7,8,9,10,11,12,13,14].

In our trial, FA doses were determined according to evidence of efficacy and recommendations in occurrence and recurrence of NTDs [26], and to results on other adverse pregnancy outcomes [27,28]. In the study, 4.0 mg of FA daily did not reduce the incidence of CMs compared to 0.4 mg daily, as suggested by a cohort-controlled trial in Hungary [2] and a case-control study in the United States [3]. However, the reader should remember that our trial was not adequately powered for this outcome, as explained before. Of note, we found no cases of trisomy 21 syndrome in the 4.0 mg FA group compared to three cases in the 0.4 mg daily. Similarly, the Hungarian study reported a 60% risk reduction of Down syndrome in women who had taken 6–9 mg/day of FA before pregnancy [38].

The role of FA supplementation in the prevention of other adverse pregnancy outcomes is still not completely clear [4,5], but previous studies observed an association between higher level of folate and fewer spontaneous abortions [28], SGA [23] and preterm birth [27]. Our results were in agreement with such findings, as 4.0 mg FA supplementation was associated with decreased occurrence of spontaneous abortion, SGA and preterm delivery when compared to 0.4 mg FA. Since adequate folate supply seems to play an important role in the implantation and development of the placenta, it could be strongly involved in this association.

The placenta develops in the periconception period and unhealthy maternal lifestyle or micronutrient deficiency such as FA can detrimentally influence placental development and function. Ahmed et al. [39] assessed the effects of increasing FA on placenta health and function across a wide range of concentrations and showed that these may be compromised in conditions of folate deficiency but not necessarily in conditions of excess FA.

According to the most common public health recommendations, we compared FA supplementation using 4.0 mg/day versus 0.4 mg/day from randomization until the 12th gestational week. In our RCT, preterm delivery and SGA, outcomes potentially linked to FA intake during pregnancy, did not significantly differ in women who continued to use FA after 12th gestational week.

Population concerns regarding FA were mainly associated with the effect of masking pernicious anemia due to B12 deficiency, to the possible effects on cancer and to potential changes in offspring neurodevelopment and epigenetic modifications [17,18,24,25].

Current exposure to FA through fortification in the United States has been reported not to increase the risk of masking anemia [40,41]. In relation to the cancer issue, Vollset et al. [42] performed collaborative meta-analyses of site-specific cancer incidence during the scheduled treatment period among 50,000 individuals from all available large cardiovascular and adenoma trials. They found that FA supplementation did not substantially increase or decrease incidence of site-specific cancer during the first 5 years of treatment. Regarding potential changes in offspring neurodevelopment and epigenetic modification, further studies are required, especially to identify the most effective dose of FA, the upper threshold of FA intake, and the ideal timing of FA supplementation for optimal neurodevelopment in humans [24,25,43,44]. 

Furthermore, folate functional insufficiency is becoming one of the most common nutritional deficiencies. The first consequence of even light folate insufficiency is a rise in plasmatic homocysteine. This condition is clinically silent but is now recognized as a metabolic risk factor for many multifactorial diseases [45,46].

Our trial has some limitations that should be considered. The study was unable to sustain the desired rate of recruitment and to achieve the estimated sample size. Despite a specific enrolment strategy, we believe that the lack of preconception counselling in Italy and routine referral setting for women planning a natural pregnancy were likely to be the causes of such a low rate in patient recruitment. Moreover, we did not assess the folate status of women by measuring serum or red blood cell folate concentrations, as well as the dietary folate intakes. In relation to these very important assessments, Italian studies conducted in blood donors reported that total B vitamin levels were lower than those considered appropriate in men and women, as were the consumption of fruits and vegetables [47,48]. Another limitation was birth data collection which was based on maternal phone interview by a trained HCP using a predefined form. However, clinical research staff obtained data on outcome measures with the collaboration of investigators and clinical centers, and the evaluation of medical records. In addition, previous studies found a good concordance between maternal recall and birth records [49]. Further, we cannot exclude that the characteristics of the selected population may not fully overlap with those of the general population, thus limiting the generalizability of the findings. Moreover, the proportion of women lost to follow-up represents another limitation of the trial. Finally, the adherence rate might have partially influenced the outcomes, but we believe that the low occurrence of the outcome measures and adverse events limited the magnitude of such influence. For all these reasons, caution is suggested when evaluating the results, which are nevertheless of interest.

## 5. Conclusions

In conclusion, this RCT failed to show any advantage of FA 4.0 mg versus 0.4 mg daily supplementation on the occurrence of CMs. However, FA 4.0 mg supplementation started before pregnancy to 12th gestational week was associated with fewer spontaneous abortions, SGA, preterm births and with a better composite outcome including adverse pregnancy outcomes.

Primary prevention of these adverse pregnancy outcomes in the population is a crucial policy priority. Adverse pregnancy outcomes such as spontaneous abortion, SGA and preterm delivery have a massive impact on health and quality of life of couples and children. Our study suggests that FA 4.0 mg supplementation starting before pregnancy may reduce such adverse pregnancy outcomes. Additional investigations are warranted to confirm our findings in subgroups of the population such as vegetarians and considering measurement of intake and status of FA, to provide useful indications for policy makers and stakeholders.

## Figures and Tables

**Table 1:** Characteristics of the Trial Participants included in the analysis.

Characteristic	4.0 mg FA Group (*n* = 227)	0.4 mg FA Group (*n* = 204)
Sociodemographic characteristics		
Maternal age, years		
Mean age (SD)	32.2 (4.4)	32.3 (4.0)
<30-no. (%)	67 (29.5)	54 (26.5)
30–34	88 (38.8)	85 (41.7)
35–39	63 (27.8)	59 (28.9)
≥40	9 (4.0)	6 (2.9)
Missing	-	-
Education, years		
≤8-no. (%)	18 (7.9)	14 (6.9)
9–13	85 (37.4)	90 (44.1)
14–18	88 (38.8)	81 (39.7)
≥19	35 (15.4)	19 (9.3)
Missing	1	-
Marital status, no. (%)		
married	160 (70.5)	146 (71.6)
common-low wife	51 (22.5)	39 (19.1)
unmarried	16 (7.0)	19 (9.3)
Lifestyle/Personal habits		
Cigarette smokers, no. (%)		
No	195 (85.9)	176 (86.3)
Yes:	32 (14.1)	28 (13.7)
≤10 cig/day	26 (11.5)	25 (12.3)
>10 cig/day	6 (2.6)	3 (1.5)
Alcohol drinkers, no. (%)		
No	120 (52.9)	95 (46.6)
Yes:	107 (47.1)	109 (53.4)
≤7 drinks/week	101 (44.5)	101 (49.5)
>7 drinks/week	6 (2.6)	8 (3.9)
BMI and current medical conditions		
BMI ^a^		
Mean (SD)	22.1 (2.8)	21.9 (2.8)
<18.50 kg/m2, no. (%)	15 (6.6)	14 (6.9)
18.50–24.99 kg/m^2^	180 (79.3)	158 (77.5)
≥25.00 kg/m^2^	32 (14.1)	32 (15.7)
Current medical conditions, no. (%)		
No	183 (80.6)	163 (79.9)
Yes:	44 (19.4)	41 (20.1)
hypertension	1 (0.4)	0 (0.0)
thyroid diseases	11 (4.8)	14 (6.9)
rheumatic diseases	1 (0.4)	2 (1.0)
urinary tract infections	3 (1.3)	3 (1.5)
genital tract infections	8 (3.5)	5 (2.5)
other diseases	24 (10.6)	21 (10.3)
Reproductive history		
Previous pregnancies, no. (%)		
0	117 (51.5)	107 (52.5)
1	72 (31.8)	67 (32.8)
≥2	38 (16.7)	30 (14.7)
Livebirth ^b^, no. (%)		
0	38 (34.5)	38 (39.2)
1	66 (60.0)	54 (55.7)
≥2	6 (5.5)	5 (5.2)
Spontaneous abortion ^b^, no. (%)		
0	50 (45.5)	44 (45.4)
1	46 (41.8)	46 (47.4)
≥2	14 (12.7)	7 (7.2)
Perinatal death ^b^, no. (%)		
No	109 (99.1)	93 (95.9)
Yes	1 (0.9)	4 (4.1)
Fetus/child with malformation or genetic disease ^b^, no. (%)		
No	108 (98.2)	94 (96.9)
Yes	2 (1.8)	3 (3.1)
Current pregnancy		
Planned pregnancy, months		
Mean (SD)	5.36 (11.1)	6.35 (11.3)
<4-no. (%)	159 (70.7)	131 (64.3)
4–7	29 (12.9)	26 (12.7)
≥8	37 (16.4)	47 (23.0)
Missing	2	-
Use of supplements before randomization, no. (%)		
No	146 (64.3)	124 (60.8)
Yes	81 (35.7)	80 (39.2)
Use of FA after the 12th week of gestation, no. (%)		
No	155 (76.7)	131 (79.4)
Yes	31 (15.4)	19 (11.5)
Missing	16 (7.9)	15 (9.1)

FA: folic acid. ^a^ Body mass index (BMI) is weight in kilograms divided by square of the height in meters (kg/m^2^). ^b^ Among women with previous pregnancies.

**Table 2:** Primary, composite, other adverse pregnancy outcomes.

Outcome	4.0 mg FA Group (*n* = 227)	0.4 mg FA Group (*n* = 204)	RR (95%CI)	*p* Value	Adjusted RR (95%CI)	*p* Value
Major CMs, no. (%)	8 (3.5)	9 (4.4)	0.80 (0.31 to 2.03)	0.64	0.76 (0.30 to 1.92) ^a^	0.56 ^a^
Composite outcome ^b^, no. (%)						
No	184 (81.1)	129 (63.2)	Ref. cat.		Ref. cat.	
Yes	43 (18.9)	75 (36.8)	0.51 (0.40 to 0.68)	<0.0001	0.52 (0.38 to 0.72) ^c^	0.0001 ^c^
Spontaneous abortion, no. (%)	21 (9.3)	34 (16.7)	0.56 (0.33 to 0.92)	0.02	0.56 (0.34 to 0.93) ^d^	0.02 ^d^
Intrauterine fetal death ^e^, no. (%)	1 (0.4)	1 (0.5)	0.90 (0.06 to 14.27)	0.94	-	-
Preeclampsia, no. (%)	2 (0.9)	3 (1.5)	0.60 (0.10 to 3.55)	0.57	-	-
Abruptio placentae, no. (%)	0 (0.0)	2 (1.0)	0.18 (0.01 to 3.72)	0.22 ^f^	-	-
SGA (<10°) ^g^, no. (%)	8 (3.5)	17 (8.3)	0.42 (0.19 to 0.96)	0.04	0.43 (0.19 to 0.97) ^a^	0.04 ^a^
Preterm delivery, no. (%)	9 (4.0)	18 (8.8)	0.45 (0.21 to 0.98)	0.04	0.44 (0.20 to 0.95) ^a^	0.04 ^a^

CMs: congenital malformations. FA: folic acid. SGA: small for gestational age. ^a^ Adjusted for FA after the 12th week of gestation. ^b^ At least one of the subsequent adverse outcomes: spontaneous abortion, intrauterine fetal death, preeclampsia, abruptio placentae, SGA, preterm delivery, major CMs. ^c^ Adjusted for maternal age, BMI, cigarette smoking, alcohol drinking, FA after the 12th week of gestation. ^d^ Adjusted for maternal age, BMI, cigarette smoking, alcohol drinking. ^e^ ≥22 weeks of pregnancy. ^f^ Fisher exact test. ^g^ Term and preterm, single and twin delivery. Adjusted analyses could not be performed due to the small occurrence of some outcome measures.

**Table 3:** Other outcomes.

Outcome	4.0 mg FA Group (*n* = 227)	0.4 mg FA Group (*n* = 204)	RR (95% CI) or MD (95% CI)	*p* Value	Adjusted RR (95% CI) or MD (95% CI)	*p* Value
Ectopic pregnancy, no. (%)	3 (1.3)	2 (1.0)	1.35 (0.23 to 7.99)	0.74	-	-
Voluntary abortion, no. (%)	1 (0.4)	1 (0.5)	0.90 (0.06 to 14.27)	0.94	-	-
LBW ^a^, no. (%)	9 (4.0)	21 (10.3)	0.39 (0.18 to 0.82)	0.01	-	-
Birthweight ^a^, mean (SD)	3357 (470.4)	3213 (597.5)	144 (34 to 254)	0.01	145 (35 to 255) ^b^	0.01 ^b^
Twin delivery, no. (%)	3 (1.3)	6 (2.9)	0.45 (0.11 to 1.77)	0.25	-	-

FA: folic acid. LBW: low birthweight. ^a^ Term and preterm, single and twin delivery. ^b^ Adjusted for folic acid after the 12th week of gestation. Adjusted analyses could not be performed due to the small occurrence of some outcome measures.

**Table 4:** Mode of delivery and admission to NICU.

Characteristic	4.0 mg FA Group	0.4 mg FA Group	*p* Value
Mode of Delivery, no. (%)	*n* = 201	*n* = 166	0.21
Spontaneous vaginal delivery	99 (50.3)	77 (47.2)	
Induced vaginal delivery	37 (18.8)	30 (18.4)	
Operative vaginal delivery	12 (6.1)	12 (7.4)	
Elective caesarean section	26 (13.2)	13 (8.0)	
Emergency caesarean section	23 (11.7)	31 (19.0)	
Missing	4	3	
Admission to NICU, no. (%)	*n* = 204	*n* = 172	0.38
No	173 (88.3)	140 (84.8)	
Yes, for observation	18 (9.2)	18 (10.9)	
Yes, for intensive care	5 (2.5)	7 (4.2)	
Missing	8	7	

## Data Availability

Data will be available upon request.

## Supplementary Materials

The following are available online at https://www.mdpi.com/article/10.3390/nu13124422/s1, Table S1: Characteristics of the Trial Participants at baseline.

## Membership of the Italian Folic Acid Trial Study Group

General Coordination Center: Renata Bortolus, Francesca Filippini, Erika Rigotti, Francesca Rech Morassutti, Antonella Noya di Lannoy (Verona University Hospital, Verona), Pierpaolo Mastroiacovo, Emanuele Leoncini (International Centre on Birth Defects -ICBD, Rome), Daniele Trevisanuto (Department of Woman’s and Child’s Health, University of Padua), Fabio Parazzini (Department of Woman, Newborn and Child, Fondazione Istituto di Ricovero e Cura a Carattere Scientifico-IRCCS Ca’ Granda Ospedale Maggiore Policlinico, Milan and Department of Clinical Sciences and Community Health, University of Milan, Milan). Experimental Medicine Management: Alessandra Compagni, Silvia Manfrè, Federica Todino, Marilisa Coati (Verona University Hospital, Verona). Randomization Centers and Study Investigators: Verona University Hospital-Borgo Roma, Verona-Giovanni Zanconato, Anna Angeli, Elena Cavaliere, Elena Mantovani; Fondazione IRCCS Cà Granda Ospedale Maggiore Policlinico, Milan-Claudia Scarduelli, Edgardo Somigliana, Federica Alagna; Gallarate Hospital-Elena Cesari; S. Bonifacio Hospital-Debora Balestreri, Elisa Caloi; Verona University Hospital-Borgo Trento, Verona-Maria Teresa Zenorini, Greta Cherubini; Vicenza Hospital-Giuliano Zanni, Chiara Vernier; Family Planning Service, Villorba-Nedelia Minisci; Family Planning Service, Padua-Anna Casaro; General Practitioner, Schio-Gian Pietro Piazza; Azienda Ospedaliera of Padua-Erich Cosmi, Silvia Visentin; Family Planning Service, Verona-Patrizia Rosi; Family Planning Service, Martellago-Maria Grazia Salviato; General Practitioner, Malo-Chiara Tresso; Thiene/Santorso Hospital-Gianfranco Jorizzo, Gaetana Cirelli; Legnago Hospital-Achille Morandini, Paola Zanini; Family Planning Service, Verona-Graziella Mortaro; Conegliano Hospital-Andrea Baffoni, Michaela Bertezzolo; Bassano del Grappa Hospital-Paola Lanza, Anna De Toni; Family Planning Service, Bassano del Grappa-Simonetta Marinangeli; General Practitioner, Verona-Laura Valotto; Luigi Sacco Hospital, Milan-Irene Cetin, Arianna Laoreti; Family Planning Service, Montebelluna-Carlo Failli; ARNAS Garibaldi Nesima Hospital, Catania-Giuseppe Ettore, Sebastiano Bianca; Bussolengo Hospital-Giorgia Negrini; General Practitioner, Sovizzo-Renato Ruffini; Family Planning Service, Vigonovo-Anna Sandri; Valdagno Hospital-Claudia Guaraldi; Family Planning Service, Mira-Marina Matterazzo; General Practitioner, Verona-Angelo Guido; General Practitioner, Dueville-Enrico Ioverno; Family Planning Service, San Pietro di Stra-Alessandra Schiavo; Adria Hospital-Enrico Di Mambro; Family Planning Service, Oderzo-Maurizia Marzolini.
