# Supplementary material for: Efficacy of 4.0 mg versus 0.4 mg Folic Acid Supplementation on the Reproductive Outcomes: A Randomized Controlled Trial
Source: Nutrients. 2021 Dec 10;13(12):4422. doi: 10.3390/nu13124422 (PMC8704306; doi:10.3390/nu13124422)
Supplement: Supplementary file 1 [file nutrients-13-04422-s001.zip › nutrients-1470719-supplementary.pdf]

Table S1. Characteristics of the Trial Participants at Baseline.

Characteristic	4 mg FA Group (*n* = 529)	0.4 mg FA Group (*n* = 531)	p-value[¥]
**Sociodemographic characteristics**			
**Maternal age** - yr			0.68
< 30 - no. (%)	125 (23.6)	130 (24.5)	
30-34	203 (38.4)	188 (35.4)	
35-39	151 (28.5)	166 (31.3)	
≥ 40	50 (9.5)	47 (8.9)	
Missing	-	-	
Mean age (SD)	33.1 (4.7)	33.0 (4.7)	
**Education** - yr			0.11
≤ 8 - no. (%)	43 (8.1)	44 (8.3)	
9-13	185 (35.0)	217 (41.1)	
14-18	225 (42.6)	212 (40.2)	
≥ 19	75 (14.2)	55 (10.4)	
Missing	1	3	
**Marital status** - no. (%)			0.79
Married	354 (67.0)	363 (68.7)	
Common-low wife	124 (23.5)	115 (21.8)	
Unmarried	50 (9.5)	50 (9.5)	
Missing	1	3	
**Lifestyle/Personal habits**			
**Cigarette smokers** - no. (%)			0.98
No	443 (83.7)	444 (83.8)	
Yes:	86 (16.3)	86 (16.2)	
≤ 10 cig/day	69	72	
> 10 cig/day	17	14	
Missing	-	1	
**Alcohol drinkers** - no. (%)			0.40
No	280 (52.9)	267 (50.4)	
Yes:	249 (47.1)	263 (49.6)	
≤ 7 drinks/week	226	240	
> 7 drinks/week	23	23	
Missing	-	1	
**BMI* and current medical conditions**			
**BMI** - no. (%)			0.46
< 18.50	35 (6.6)	39 (7.4)	
18.50-24.99	405 (76.6)	415 (78.4)	
≥ 25.00	89 (16.8)	75 (14.2)	
Missing	-	2	
Mean (SD)	22.2 (2.9)	21.9 (2.9)	
**Current medical conditions** - no. (%)			0.55
No	427 (80.7)	420 (79.2)	
Yes:	102 (19.3)	110 (20.8)	

hypertension	6 (1.1)	9 (1.7)	0.43
thyroid diseases	36 (6.8)	34 (6.4)	0.79
rheumatic diseases	4 (0.8)	4 (0.8)	0.99
urinary tract infections	7 (1.3)	9 (1.7)	0.61
genital tract infections	13 (2.5)	14 (2.6)	0.84
other diseases	49 (9.3)	52 (9.8)	0.77
Missing	-	1	
**Reproductive history**			
**Previous pregnancies** - no. (%)			0.88
0	309 (58.4)	306 (57.7)	
1	146 (27.6)	153 (28.9)	
≥ 2	74 (14.0)	71 (13.4)	
Missing	-	1	
**Livebirth**** - no. (%)			0.37
0	88 (40.0)	92 (41.1)	
1	121 (55.0)	114 (50.9)	
≥ 2	11 (5.0)	18 (8.0)	
**Spontaneous abortion**** - no. (%)			0.20
0	106 (48.2)	110 (49.1)	
1	85 (38.6)	95 (42.4)	
≥ 2	29 (13.2)	19 (8.5)	
**Perinatal death**** - no. (%)			0.24
No	215 (97.7)	222 (99.1)	
Yes	5 (2.3)	2 (0.9)	
**Fetus/child with malformation or genetic disease**** - no. (%)			0.82
No	213 (96.8)	216 (96.4)	
Yes	7 (3.2)	8 (3.6)	
**Use of supplements before randomization** - no. (%)			0.58
No	342 (64.7)	351 (66.2)	
Yes:	187 (35.3)	179 (33.8)	
folic acid	114 (21.6)	106 (20.0)	0.52
folic acid and multivitamins	26 (4.9)	17 (3.2)	0.15
multivitamins without folic acid	47 (8.9)	56 (10.6)	0.48
missing	-	1	

* The body-mass index (BMI) is the weight in kilograms divided by the square of the height in meters;

** among women with previous pregnancies

¥ Chi-square p-value
